# The Influence of Reinforced Fibers and Opacifiers on the Effective Thermal Conductivity of Silica Aerogels

**DOI:** 10.3390/gels10050300

**Published:** 2024-04-26

**Authors:** Binghuan Huang, Jingbei Li, Liang Gong, Pengcheng Dai, Chuanyong Zhu

**Affiliations:** 1 College of New Energy, China University of Petroleum (East China), Qingdao 266580, China; 2 College of Chemistry and Chemical Engineering, China University of Petroleum (East China), Qingdao 266580, China

**Keywords:** fiber–particle-reinforced silica aerogels, effective thermal conductivity, radiative characteristics, high temperature

## Abstract

Fiber–particle-reinforced silica aerogels are widely applied in thermal insulation. Knowing their effective thermal conductivity (ETC) and radiative characteristics under high temperatures is necessary to improve their performance. This article first analyzes the radiation characteristics of silica aerogels doped with opacifier particles and reinforced fibers, and then a universal model is established to predict the ETC. Furthermore, the impacts of different parameters of opacifier particles and reinforced fibers on the thermal insulation performance of silica aerogels are investigated. The results indicate that SiC exhibits comparatively strong absorption characteristics, making it a good alternative for opacifiers to improve thermal insulation performance under high temperatures. For the given type and volume fraction of opacifier particles, there exists an optimal diameter and volume fraction to achieve the best insulation performance of silica aerogel under a certain temperature. Considering that SiO_2_ fibers exhibit a limited extinction capability and higher conductive thermal conductivity under high temperatures, for fiber–particle-reinforced silica aerogels, it is beneficial for their insulation performance to reduce the fiber volume fraction when the required mechanical properties are satisfied.

## 1. Introduction

Silica aerogel is considered to be an important solid material [1,2] for its lightweight and low thermal conductivity, and is regarded as a promising insulation material. However, pure silica aerogels suffer the disadvantages of brittleness and infrared transmittance [3], resulting in difficulties in maintaining their structural integrity and high-intensity radiation heat transfer under high-temperature circumstances. In order to address these problems, fibers are doped into silica aerogels to promote their mechanical performance, while opacifiers are mixed into silica aerogels to improve their thermal insulation performance under high temperatures, thus, a fiber–particle-reinforced hybrid composite [4,5] is obtained. However, due to the mixture of the reinforced composites, the heat conduction and radiative heat transfer characteristics in such materials are much more complicated than those in the pure matrix, and the different effects of fibers and particles in the conduction and the radiation pose a more significant challenge in predicting the effective thermal conductivity (ETC) of composites reinforced with fibers and particles.

Over the past decades, scholars have conducted many efforts to establish models to describe the heat transfer characteristics of different types of multi-composites, including particle-reinforced composites [6], fiber-reinforced composites [7,8], thin-layer-fillers-reinforced composites [9], and foam-reinforced composites [10] through different methods. For instance, Gonzo [6] developed two correlations for predicting the ETC of particle-reinforced composites based on Maxwell’s model and Chiew and Glandt’s model [11,12]. They stated that the correlations proposed were available for predicting the thermal conductivity of particle-reinforced composites within an extensive range. Yan et al. [8] concentrated on a unit cell and adopted an eigenfunction expansion–variational method to investigate the ETC of double-periodic fiber-reinforced composites. Thanks to their contributions, the heat conduction mechanisms of various multi-composites were gradually revealed, and corresponding ETC models have been developed. However, these studies did not take the effect of reinforced fibers and opacifier particles on radiative heat transfer into consideration.

There exist many coupling models proposed by scholars to predict the thermal conductivity of a silica aerogel matrix, including the fractal model, the unit model, and the numerical model [13,14]. Xie et al. [15] proposed their fractal model by combining a Sierpinski sponge structure with an intersected sphere structure, and this new structure was utilized to represent the fundamental frame of silica aerogels. Spagnol et al. [16] adopted different two-dimensional fractal structures to approximate the frames of silica aerogels, then they calculated their corresponding thermal conductivity based on their approximate structure. Zeng et al. [17] developed a series of models for predicting the thermal conductivity of a silica aerogel matrix. In these models, the unit cells of silica aerogels were assumed to be cubic arrays which are composites of intersected square pillars, intersected cylindrical pillars, or intersected spheres, respectively. However, when reinforced fibers and opacifier particles are added to silica aerogels, the heat conduction via the solid phase of silica aerogels will increase. For silica aerogels doped with spherical opacifiers, the corresponding conductive thermal conductivity of silica aerogels *λ*_cd_ can be predicted with the traditional theory of Maxwell’s equation [11]. For the ETC of pure silica aerogels doped with fibers, if the distribution of fibers is random, then the ETC can be predicted by Hamilton’s model [5]. In order to predict the ETC of silica aerogels with complex reinforced composites, scholars have conducted further studies to explore new methods. Chen et al. [18] proposed a method to estimate the ETC of fiber–opacifier-reinforced silica aerogels. Firstly, they considered the silica aerogels and the opacifiers as an effective matrix, and the corresponding ETC was calculated by Maxwell’s model. Secondly, they calculated the ETC of randomly distributed fibers and this effective matrix based on Hamilton’s model. Their efforts provide practical approaches to estimating the ETC of silica aerogels without the influence of radiation. However, as mentioned above, for high temperatures, the radiation heat transfer cannot be neglected for silica aerogels, which would significantly affect their ETC [19].

According to these analyses, it can be realized that, after being doped into reinforced fibers and opacifier particles, the thermal conductivity for silica aerogels will be increased, while the radiative heat transfer under high temperatures will be reduced. Therefore, adding fibers and opacifier particles will result in more complex characteristics in the process of heat transfer. However, the quantitative investigation into the effects of reinforced fibers and opacifier particles, especially the combination effect of these two composites, on the ETC of silica aerogels is insufficient. Motivated by this, this paper attempts to construct a model for predicting the ETC of fiber–particle-reinforced silica aerogels and quantitatively estimate the effect of reinforced fibers and opacifier particles on conductive and radiative heat transfer. This paper is organized as follows: in Section 2, the main theories of the radiative heat transfer of particles and fibers will be introduced and the prediction method of the ETC for silica aerogels will be discussed. In Section 3, the model for predicting the ETC of fiber–particle-reinforced silica aerogels will be constructed and validated. In Section 4, a quantitative analysis of the effects of reinforced fibers and opacifier particles on radiative and effective thermal conductivity will be conducted. Finally, the main conclusions will be summarized in Section 3.

## 2. Results and Discussion

### 2.1. Validation of Model

**Figure 1:**
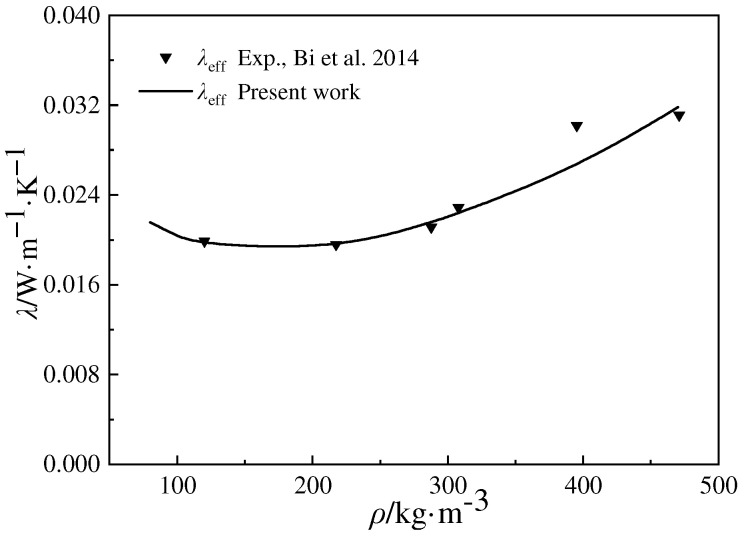
Variation in ETC of pure silica aerogels versus density (experimental data source from Ref. [20]).

As the model of ETC proposed in this paper is a universal model, its results are first compared with experimental data to verify its precision. Figure 1 shows the variation in the thermal conductivity of pure silica aerogels with an increase in density under room temperature and atmospheric pressure. The experimental data of six different samples are obtained from Ref. [20]. Samples of silica aerogels are prepared based on a sol–gel process and supercritical evaporation. The density, porosity, mean pore size, and particle size of the samples are listed in Table 1. The ETC data for all the samples are measured through the hot-plate method, which is based on one-dimensional steady-state heat conduction. Two identical samples are symmetrically attached on either side of the plate. The hot plate is a heat source to heat the samples, and two outer layers are arranged on the samples to cool their outer sides and create a temperature difference between the inner and outer sides of the sample. Due to the heat flux through the samples, the temperature difference between both sides of the sample and the thickness of the sample can be measured, and the thermal conductivity can be calculated with Fourier’s Law. It is indicated that the thermal conductivity of the pure silica aerogels obtained with the proposed model is in good agreement with the experimental results from Ref. [20].

**Figure 2:**
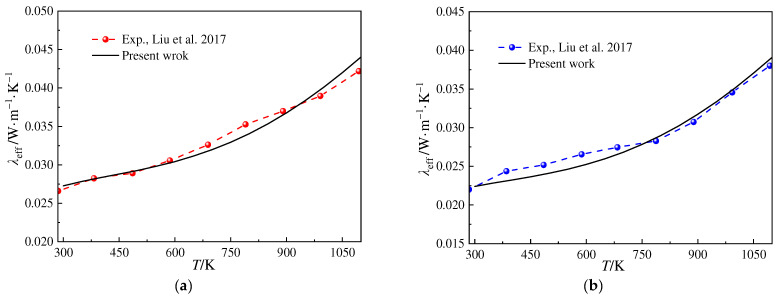
Variation in the ETC of fiber–particle-reinforced silica aerogels versus temperature (experimental data source from Ref. [21]). (**a**) *P* = 100 kPa and (**b**) *P* = 50 kPa.

Figure 2 illustrates the variation in the ETC versus temperature for the fiber–particle-reinforced silica aerogels at different atmospheric pressures. The results of the proposed model are compared with the experimental data of Ref. [21]. The porosity of the fiber–particle-reinforced silica aerogel samples is 83.2%, while the volume fractions of the solid backbone, SiO_2_ fibers, and SiC opacifier particles are 12.3%, 0.6%, and 3.9%, respectively. The average diameters of the fibers and opacifier particles are approximately 7.0 μm and 3.0 μm, respectively. The surface area of the samples is approximately estimated with the correlation formula *S* = 787.77 − 1.6812*ρ*_a_ mentioned in Ref. [22], where *ρ*_a_ represents the density of the aerogel matrix. The complex refractive indices of SiC and SiO_2_ are obtained from Refs. [19,23], respectively. In Figure 2, a good agreement can be observed between the results of the proposed model and the experimental results of reference [21]. Thus, the model of ETC used in this paper is validated, and further investigation could be conducted to discuss the heat transfer characteristics of ETC for fiber–particle-reinforced silica aerogels. 

### 2.2. Effect of Different Opacifier Particles

**Figure 3:**
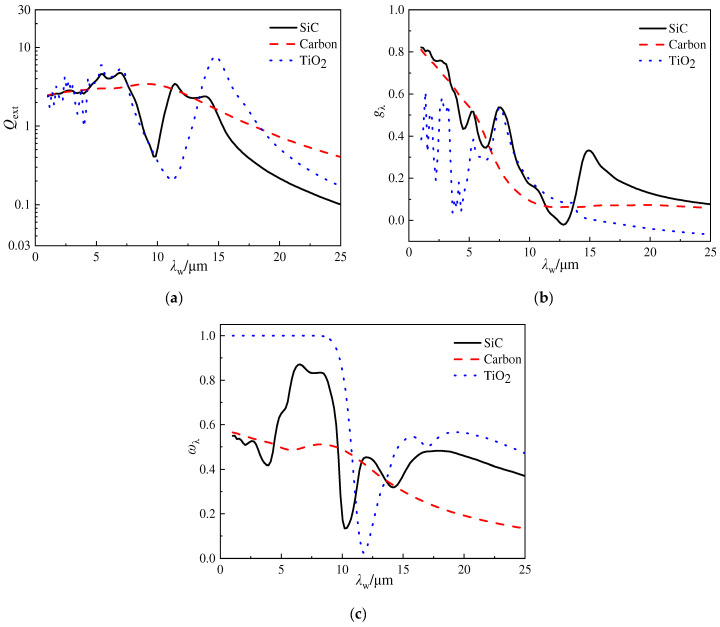
Variations in the extinction efficiency, asymmetry factor, and scattering albedo versus the wavelength for different opacifier particles. (**a**) *Q*_ext_ vs. *λ*_w_; (**b**) *g*_λ_ vs. *λ*_w_; and (**c**) *ω*_λ_ vs. *λ*_w_.

Figure 3 depicts the variations in the extinction efficiency, asymmetry factor, and scattering albedo for different opacifier particles, including SiC, carbon, and TiO_2_, versus the wavelength. The properties of the materials are listed in Table 2. The particle diameter is 3.0 μm, and the complex refractive indices of carbon and TiO_2_ are obtained from Ref. [20] and [24], respectively. From Figure 3a, it can be realized that, within the studied wavelength range, all these three types of opacifier particles exhibit a general trend of a decreasing extinction efficiency with an increase in wavelength. Additionally, for the wavelength ranges from 1.0 μm to 5.0 μm, the extinction efficiencies of these opacifier particles are approximate. When the wavelength increases from 7.5 μm to 11.0 μm, carbon shows a significantly higher extinction efficiency than the other two particles. Moreover, when the wavelength is beyond 15.0 μm, the extinction efficiencies of all the types of particles decrease rapidly with the increment in wavelength.

In Figure 3b, the asymmetry factors of SiC and carbon particles are mostly greater than zero within the investigated wavelength range, indicating predominantly forward scattering characteristics. However, for TiO_2_ particles, the asymmetry factor is negative within the wavelength ranges from 15.0 μm to 25.0 μm, suggesting backward scattering characteristics.

Figure 3c demonstrates that, within the wavelength range below 10.0 μm, the scattering albedo of TiO_2_ particles is close to 1.0, suggesting that, for TiO_2_ particles, scattering plays a predominant role in infrared radiation extinction at a shorter wavelength. Additionally, within the investigated wavelength range, the scattering albedo of TiO_2_ is consistently higher than that of SiC and carbon particles. For SiC particles, scattering is the dominant effect for wavelengths below 10.0 μm, while absorption gradually becomes more important with an increase in wavelength. In contrast, for carbon, absorption is the dominant effect throughout the entire wavelength range researched.

Therefore, different opacifier particles exhibit different mechanisms for infrared radiation extinction. Furthermore, through the analysis above, it is noted that the asymmetry factors of opacifier particles are non-zero, indicating significant anisotropic scattering characteristics. When calculating the ETC of silica aerogels doped with opacifier particles, it is necessary to transform them into isotropic scattering media to avoid errors.

**Figure 4:**
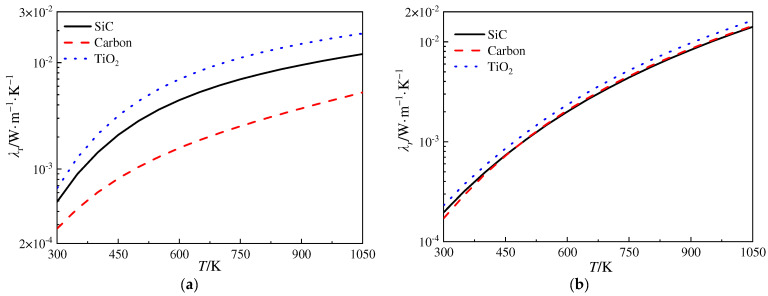
Variation in radiative thermal conductivity versus temperature for different opacifier particles. (**a**) *D*_op_ = 1.0 μm and (**b**) *D*_op_ = 3.0 μm.

Figure 4 depicts the variation in the radiative thermal conductivity versus temperature for the silica aerogels doped with opacifier particles, and for the silica aerogel matrix, the density is 220 kg·m^−3^. It is illustrated in Figure 4 that the radiative thermal conductivity of the silica aerogel increases rapidly with temperature according to Equation (22). From Figure 4a, it is plotted that, when the diameter of the particle is 1.0 μm, the radiative thermal conductivity of the silica aerogels doped with carbon is the lowest, indicating the best extinction performance. This can be attributed to the higher refractive index and absorption efficiency of carbon. However, carbon is easily oxidized at temperatures exceeding 600 K, which results in a decrease in extinction capability; hence, it is unsuitable to be adopted as the opacifier material for high temperatures. On the other hand, SiC exhibits relatively strong absorption characteristics, thus, its performance as an opacifier particle is superior to that of TiO_2_. In Figure 4b, it is noted that, when the diameter of the particle is 3.0 μm, both carbon and SiC demonstrate better extinction performances than that of TiO_2_. Furthermore, the deviation in the extinction performances of SiC and carbon is smaller than that plotted in Figure 4a, indicating that the diameter of particles is also a significant factor influencing the extinction performance of opacifiers.

### 2.3. Effect of Different Diameters for Opacifier Particles

**Figure 5:**
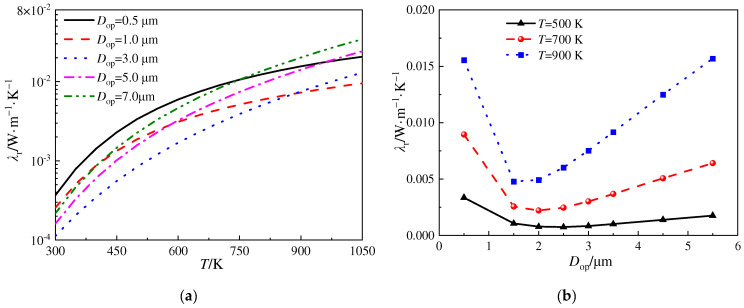
Variation in radiative thermal conductivity for different diameters of opacifier particles. (**a**) *λ*_r_ vs. *T* and (**b**) *λ*_r_ vs. *D*_op_.

Figure 5 illustrates the variation in radiative thermal conductivity versus temperature for the silica aerogels mixed with SiC opacifier particles of different diameters. The volume fraction is 3.0%. In Figure 5a, it is observed that, for temperatures below 600 K, opacifier particles of diameters of 3.0 μm and 5.0 μm exhibit relatively good extinction performances. However, as the temperature increases to approximately 780 K, opacifier particles with a diameter of 1.0 μm demonstrate the best extinction performance. Therefore, although the type and volume fraction of these opacifier particles are the same, there exists an optimal diameter to achieve the best extinction performance, which depends on the temperature. Figure 5b presents the relationship between the radiative thermal conductivity of the silica aerogels and the diameter of the SiC particles at different temperatures. It can be observed that, at 500 K, SiC particles with a diameter ranging from 2.5 to 3.0 μm exhibit the best extinction performance, while at 700 K and 900 K, SiC particles with a diameter of around 2.0 μm demonstrate the best extinction performance.

### 2.4. Effect of Different Volume Fractions for Opacifier Particles

**Figure 6:**
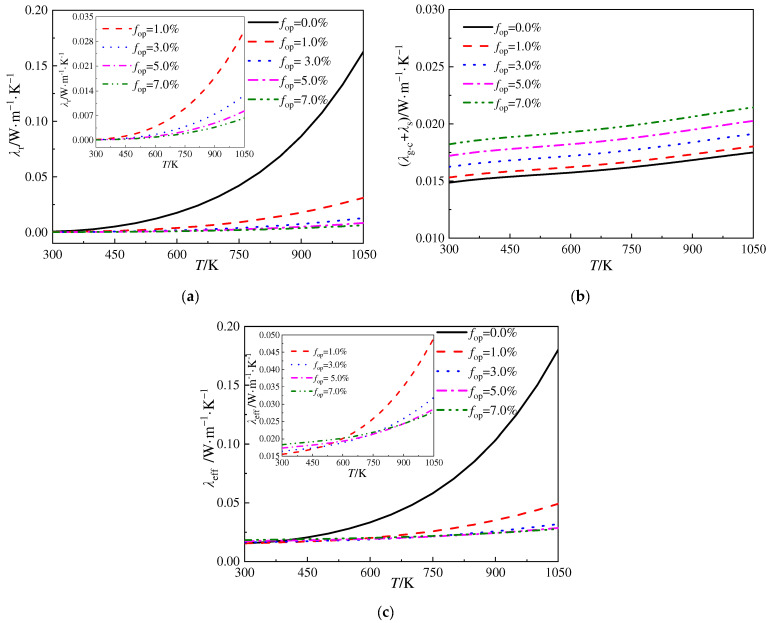
Variation in radiative thermal conductivity, the conductivity contributed by thermal conduction (*λ*_g-c_ + *λ*_s_), and the ETC versus the volume fraction of SiC opacifier particles. (**a**) *λ*_r_ vs. *T*; (**b**) *λ*_g-c_ + *λ*_s_ vs. *T*; and (**c**) *λ*_eff_ vs. *T*.

Figure 6a–c depict the variation in radiative thermal conductivity (*λ*_r_), the conductivity contributed by thermal conduction (*λ*_g-c_ + *λ*_s_), and the ETC (*λ*_eff_) versus the volume fraction of SiC opacifier particles in silica aerogels, where the diameter of the opacifier particles is 3.0 μm. From Figure 6a, it can be observed that, once the volume fraction of the particles reaches 1.0%, there is a significant reduction in the radiative thermal conductivity of the silica aerogels. With a further increment in the volume fraction of the opacifier particles, the radiative thermal conductivity gradually decreases, and the decreasing trend becomes less apparent. Figure 6b indicates that, with the increase in temperature, the values of *λ*_g-c_ + *λ*_s_ increase approximately linearly, while a larger volume fraction of the particles results in a higher value of *λ*_g-c_ + *λ*_s_. In Figure 6c, the variation in the ETC versus the volume fraction of the particles is not monotonous. This is related to the results illustrated in Figure 6a,b, where, for constant temperature conditions, the increase in the volume fraction of the particles leads to an increase in (*λ*_g-c_ + *λ*_s_) and a decrease in *λ*_r_. Therefore, attention should be paid to the fact that merely increasing the volume fraction of the particles cannot achieve the best ETC.

### 2.5. Effect of Reinforced Fibers

**Figure 7:**
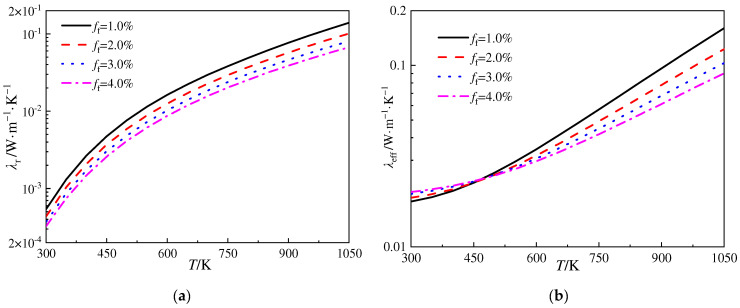
Variation in radiative thermal conductivity and ETC versus temperature for silica aerogels doped with different volume fractions of SiO_2_ fibers. (**a**) *λ*_r_ vs. *T* and (**b**) *λ*_eff_ vs. *T*.

Figure 7a,b illustrate the variation in radiative thermal conductivity and the corresponding ETC versus temperature for the silica aerogels mixed with different volume fractions of SiO_2_ fibers, respectively. The diameter of the fibers is *D*_f_ = 6.0 μm. The simulation results indicate that, as the volume fraction of the fibers increases, the radiative thermal conductivity of the silica aerogels gradually decreases. However, the values of the radiative thermal conductivity are significantly higher than those of the silica aerogels doped with SiC opacifier particles shown in Figure 6a, while the volume fractions of the reinforced fibers and opacifier particles are the same. This suggests that although the SiO_2_ fibers in this scale exhibit certain extinction performances, they are inferior to those of SiC particles. The curves in Figure 7b demonstrate that, when the temperature is lower than approximately 460 K, the increase in the volume fraction of the SiO_2_ fibers leads to an increase in the ETC of the silica aerogels, and when the temperature exceeds approximately 460 K, the ETC decreases with the augmentation of the volume fraction of the fibers. This phenomenon is due to the extinction capability of SiO_2_ fibers. As the volume fraction of the fibers continuously increases, the radiative heat transfer is further restrained. However, the conductive thermal conductivity of the SiO_2_ fibers is also larger than that of the silica aerogel matrix, thus, increasing the volume fraction of the SiO_2_ fibers would significantly enhance the thermal conductivity thanks to the promotion of conduction.

**Figure 8:**
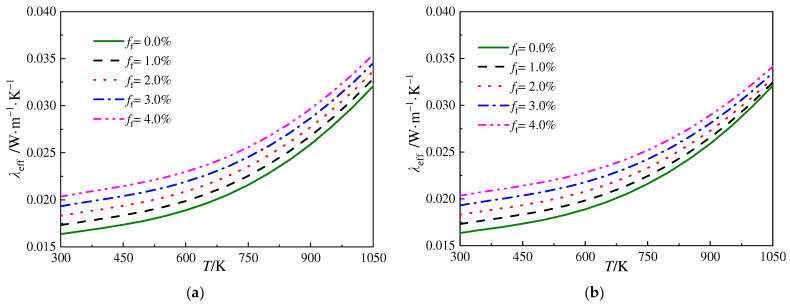
Variation in ETC versus the temperature for silica aerogels doped with SiO_2_ fibers and SiC opacifier particles. (**a**) *D*_f_ = 6.0 μm and (**b**) *D*_f_ = 3.0 μm.

Figure 8a,b plot the variation in ETC versus the temperature for the silica aerogels doped with SiO_2_ fibers and SiC opacifier particles. The diameter of the opacifier particles is 3.0 μm and the volume fraction is 3.0%, while the diameters of the fibers are 6.0 μm and 3.0 μm, respectively, and for the same diameter of fibers, the volume fraction of these fibers varies from 0.0% to 4.0%. The results indicate that, for the fiber–particle-reinforced silica aerogels within this investigation, for a certain diameter of fibers, the ETC increases monotonously with an increment in the volume fraction of the fibers. This is because, although SiO_2_ fibers exhibit a certain extinction capability, they significantly enhance the conductive thermal conduction in silica aerogels. The strengths and weaknesses of different fiber reinforcement strategies are compared in Table 3. In fiber–particle-reinforced silica aerogels, the radiative heat transfer is mainly restrained by the opacifier particles doped in the silica aerogels, and a further increment in the volume fraction of the fibers would show less effect on suppressing the radiative thermal conduction but significantly enhance the conductive thermal conduction in the solid phase; thus, the insulation performance of the silica aerogel composite would deteriorate. Therefore, for silica aerogels with a given volume fraction of opacifier particles, it is advantageous in improving their insulation performance to minimize the volume fraction of the fibers if the demands of the mechanical properties are satisfied.

## 3. Conclusions

In this paper, a model is proposed for predicting the ETC of fiber–opacifier silica aerogels, and the model is validated by comparing the results with available experimental data. Based on this model, the effects of various parameters such as the type, volume fraction, and particle size of the opacifiers, as well as those of the fibers, on the insulation characteristics of the silica aerogels are investigated. The main conclusions can be summarized as follows:

(1) For a specific type and volume fraction of opacifier particles, an optimal particle diameter exists that maximizes the extinction performance and minimizes the radiative thermal conductivity, and this optimal size is temperature-dependent. Within the temperatures investigated, opacifier particles with smaller diameters demonstrate superior extinction capabilities.
(2) The mechanisms of opacifiers for blocking radiative heat transfer performed by diverse opacifiers differ distinctly: carbon principally relies on absorptive properties, whereas TiO_2_ predominantly utilizes scattering mechanisms, and for SiC, the dominant mechanism depends on the wavelength. Furthermore, all three particles demonstrate significant anisotropic scattering properties.
(3) For the conditions and opacifier particles investigated in this paper, as the volume fraction increases to 1%, the radiative heat transfer of silica aerogel can be significantly restrained at high temperatures, which apparently improves the thermal insulation performance of the material. However, as the volume fraction further increases, the decrease in the radiative thermal radiation conductivity becomes less, while the thermal conductivity due to conduction (*λ*_g-c_ + *λ*_s_) gradually increases. Thus, for opacifier particles, there exists an optimal volume fraction that would minimize the ETC of silica aerogels.
(4) Besides opacifier particles, SiO_2_ fibers can also restrain radiative heat transfer to reduce the ETC of silica aerogels at high temperatures. However, increasing the volume fraction of SiO_2_ fibers will significantly raise the ETC of silica aerogels at low temperatures due to their higher conductive thermal conductivity compared with that of the solid backbone. Thus, it is advantageous for insulation performance to minimize the volume fraction of reinforced fibers if the demands of the mechanical properties are satisfied.

## 4. Materials and Methods

### 4.1. The Scattering Characteristics of Opacifier Particles

The absorption and scattering characteristics of opacifier particles on infrared light are the basis for investigating the radiative heat transfer of opacifier particles and that of silica aerogel composite materials. As indicated in previous studies, the scattering characteristics of opacifier particles can be described by Mie theory [1,16]. According to the Mie theory, for a single spherical opacifier particle, it can be derived as [25]

(1) $$Q_{\mathrm{sca},\mathrm{op}}=\frac{2}{x^{2}}\overset{\infty }{\underset{n=1}{\sum }}(2n+1)\left({\left|a_{n}\right|}^{2}+{\left|b_{n}\right|}^{2}\right)$$

(2) Qext,op=2x2Re[∑n=1∞(2n+1)an+bn]

(3) $$Q_{\mathrm{abs},\mathrm{op}}=Q_{\mathrm{ext},\mathrm{op}}-Q_{\mathrm{sca},\mathrm{op}}$$

(4) $$\omega_{\lambda }=\frac{Q_{\mathrm{sca},\mathrm{op}}}{Q_{\mathrm{ext},\mathrm{op}}}$$

where *Q*_sca,op_ refers to the scattering efficiency and *Q*_ext,op_ refers to the extinction efficiency, while *Q*_abs,op_ refers to the absorption efficiency. *x* represents the size parameter related to the diameter of the opacifier particle *D*_op_ and the wavelength of infrared light *λ*_w_, which is defined as *x* = 2*πD*_op_/*λ*_w_. *ω* is the scattering albedo of opacifier particles. *Re* is the real part of a complex quantity and *a_n_* and *b_n_* represent the Mie coefficients, which are defined as follows:

(5) $$a_{n}=\frac{\psi_{n}\left(x\right)\psi_{n}’\left(mx\right)-m\psi_{n}’\left(x\right)\psi_{n}\left(mx\right)}{\xi_{n}\left(x\right)\psi_{n}’\left(mx\right)-m\xi_{n}’\left(x\right)\psi_{n}\left(mx\right)}$$

(6) $$b_{n}=\frac{m\psi_{n}\left(x\right)\psi_{n}’\left(mx\right)-\psi_{n}’\left(x\right)\psi_{n}\left(mx\right)}{m\xi_{n}\left(x\right)\psi_{n}’\left(mx\right)-\xi_{n}’\left(x\right)\psi_{n}\left(mx\right)}$$

(7) $$\xi_{n}=\psi_{n}+i\chi_{n}$$

where *m* is the complex refractive index of the opacifier particles, and *ψ_n_* and *χ_n_* represent the Bessel function and satisfy:

(8) $$\psi_{n+1}\left(x\right)=\frac{2n+1}{x}\psi_{n}\left(x\right)-\psi_{n-1}\left(x\right),\chi_{n+1}\left(x\right)=\frac{2n+1}{x}\chi_{n}\left(x\right)-\chi_{n-1}\left(x\right)$$

(9) $$\psi_{-1}\left(x\right)=\cos x,\psi_{0}\left(x\right)=\sin x,\chi_{-1}\left(x\right)=c-\sin x,\chi_{0}\left(x\right)=\cos x$$

The asymmetry factor of a spherical opacifier particle in scattering can be expressed as:

(10) gλ=4x2Qsca∑n=1∞nn+2n+1Re(anan+1∗+bnbn+1∗)+2n+1nn+1Re(anbn∗)

For a single opacifier particle, the corresponding extinction efficiency can be obtained with Equation (2), and the spectral extinction coefficient of all opacifier particles can be approximately calculated as [26]

(11) $$\beta_{\mathrm{op},\lambda }=\frac{3}{2}\frac{f_{\mathrm{op}}Q_{\mathrm{ext},\mathrm{op}}}{D_{\mathrm{op}}}$$

where *f*_op_ refers to the volume fraction of the opacifier particles. Considering the anisotropic scattering characteristics of opacifier particles, it is necessary to use the transport approximation to equate them to an isotropic scattering medium. The transport spectral extinction coefficient of the opacifier particles can be expressed as [22]

(12) $$\beta_{\mathrm{op},\lambda }^{\mathrm{tr}}=\beta_{\mathrm{op},\lambda }\left(1-\omega_{\lambda }g_{\lambda }\right)$$

### 4.2. The Scattering Characteristics of Reinforced Fibers

The absorption and scattering characteristics of reinforced fibers can also affect the radiative heat transfer characteristics in silica aerogels. The scattering performance of a single reinforced fiber can be described by the Mie theory, as the fiber can be treated as long cylinders. The extinction efficiency and scattering efficiency of reinforced fibers are expressed as follows [25]:

(13) Qext,fϕ=1xRe[a02+b01+2∑n=1∞an2+bn1]

(14) $$Q_{\mathrm{sca},f}\left(\phi \right)=\frac{1}{x}\left[{\left|a_{02}\right|}^{2}+{\left|b_{01}\right|}^{2}+2\overset{\infty }{\underset{n=1}{\sum }}\left({\left|a_{n1}\right|}^{2}+{\left|a_{n2}\right|}^{2}+{\left|b_{n1}\right|}^{2}+{\left|b_{n2}\right|}^{2}\right)\right]$$

where *x* represents the size parameter related to the diameter of the reinforced fiber *D*_f_ and the wavelength of infrared light *λ*_w_, which is defined as *x* = 2*πD*_f_/*λ*_w_. *ϕ* is the incident angle of infrared light, and *a_n_*_1_, *a_n_*_2_, *b_n_*_1_, and *b_n_*_2_ can be expressed as functions of the incident angle and fiber optical parameters [25].

As the scattering characteristics of the reinforced fibers are related to their distribution directions, assuming that the reinforced fibers are uniformly distributed in the silica aerogels, to obtain the equivalent extinction and scattering factors, integration over the whole range of the incident angle is necessary, as:

(15) $${\bar{Q}}_{\mathrm{ext},f}=\frac{2}{\pi }\int_{0}^{\frac{\pi }{2}}Q_{\mathrm{ext},f}\left(\phi \right)d\phi$$

(16) $${\bar{Q}}_{\mathrm{sca},f}=\frac{2}{\pi }\int_{0}^{\frac{\pi }{2}}Q_{\mathrm{sca},f}\left(\phi \right)d\phi$$

Taking the anisotropic scattering characteristics of the reinforced fibers into account and using the transport approximation to equate them to an isotropic scattering medium, the transport spectral extinction coefficient of the reinforced fibers can be calculated as:

(17) $${\bar{Q}}_{\mathrm{ext},f}^{\mathrm{tr}}=\frac{2}{\pi }\int_{0}^{\frac{\pi }{2}}Q_{\mathrm{ext},f}\left(\phi \right)\left(1-\omega \left(\phi \right)g\left(\phi \right)\right)d\phi$$

where *g*(*ϕ*) is the asymmetry factor of the reinforced fibers. For reinforced fibers in a diameter of *D_f_* with a volume fraction of *f*_f_, the transport spectral extinction coefficient can be expressed as [15]:

(18) $$\beta_{\lambda ,f}^{\mathrm{tr}}=\frac{4{\bar{Q}}_{\mathrm{ext},f}^{\mathrm{tr}}f_{f}}{\pi D_{f}}$$

### 4.3. The Radiative Thermal Conductivity for Fiber–Particle-Reinforced Silica Aerogels 

The radiative heat transfer process in fiber–particle-reinforced silica aerogels can be treated as a diffusion process, because they are usually considered as optical thickness materials. Then, the radiation flux can be calculated according to Rosseland diffusion approximation, as follows [3]:

(19) $$q_{r}=-\frac{16n_{\mathrm{ref}}^{2}}{3\beta_{\mathrm{total}}\left(T\right)}\sigma T^{3}\frac{\partial T}{\partial x}$$

where *σ* represents the Stefan-Boltzmann constant and *n*_ref_ refers to the refractive index of the fiber–particle-reinforced silica aerogels. *β*_total_(*T*) is the Rosseland mean extinction coefficient, which is a function that depends on temperature, and it can be estimated as [3]

(20) $$\beta_{\mathrm{total}}\left(T\right)={\left[\int_{0}^{\infty }\frac{1}{\beta_{\lambda }^{\mathrm{tr}}}\frac{\partial E_{b\lambda }}{\partial E_{b}}d\lambda \right]}^{-1}$$

where $\beta_{\lambda }^{\mathrm{tr}}$ is the transport spectral extinction coefficient of the fiber–particle-reinforced silica aerogels, and can be described as:

(21) $$\beta_{\lambda }^{\mathrm{tr}}=\beta_{\lambda ,\mathrm{op}}^{\mathrm{tr}}+\beta_{\lambda ,f}^{\mathrm{tr}}+\left(1-f_{\mathrm{op}}-f_{f}\right)\beta_{\lambda ,a}$$

where *β*_λ,a_ refers to the spectral extinction coefficient of silica aerogels [27].

Based on the Rosseland diffusion approximation, the radiative thermal conductivity of a silica aerogel doped with fibers and opacifier particles can be expressed as follows [3]:

(22) $$\lambda_{r}=\frac{16n_{\mathrm{ref}}^{2}}{3\beta_{\mathrm{total}}\left(T\right)}\sigma T^{3}$$

### 4.4. The ETC for Fiber–Particle-Reinforced Silica Aerogels

As mentioned above, the ETC for fiber–particle-reinforced silica aerogels *λ*_eff_ can be expressed as:

(23) $$\lambda_{\mathrm{eff}}=\lambda_{\mathrm{cd}}+\lambda_{r}$$

where *λ*_cd_ is the conductive thermal conductivity and *λ*_r_ is the radiative thermal conductivity. *λ*_cd_ is a function that can be expressed as:

(24) $$\lambda_{\mathrm{cd}}=f\left(\lambda_{a},\lambda_{\mathrm{op}},\lambda_{f},f_{\mathrm{op}},f_{f}\right)$$

where *λ*_a_, *λ*_op_, and *λ*_f_ are the thermal conductivity of the silica aerogels matrix, opacifier particles, and reinforced fibers, respectively. *f*_op_ and *f*_f_ are the volume fractions of the opacifier particles and reinforced fibers.

(25) $$\lambda_{a}=\lambda_{s}+\lambda_{g-c}$$

where *λ*_s_ is the thermal conductivity of the silica solid backbone and *λ*_g-c_ is the gas-contributed thermal conductivity; they can be estimated by the theories from Ref. [28].

The calculation of *λ*_cd_ can be divided into two steps. Firstly, assuming that the silica aerogels are only reinforced with opacifier particles, the corresponding conductive thermal conductivity *λ*_a-o_ can be calculated as [25]:

(26) $$\lambda_{a-o}=\left(1+\frac{3(\lambda_{o}/\lambda_{a}-1)f_{o}}{\left(\lambda_{\mathrm{op}}/\lambda_{a}+2\right)-\left(\lambda_{o}/\lambda_{a}-1\right)f_{o}}\right)\lambda_{\mathrm{op}}$$

Secondly, assuming the fibers are doped into the silica aerogels above to obtain the fiber–particle-reinforced silica aerogels, the conductive thermal conductivity *λ*_cd_ can be expressed as [25]

(27) $$\lambda_{\mathrm{cd}}=\left(\frac{\lambda_{f}/\lambda_{a-o}+\left(n-1\right)+\left(n-1\right)\left(\lambda_{f}/\lambda_{a-o}-1\right)f_{f}}{\lambda_{f}/\lambda_{a-o}+\left(n-1\right)+\left(1-\lambda_{f}/\lambda_{a-o}\right)f_{f}}\right)\lambda_{a-o}$$

where *n* is the shape parameter and *n* = 6 for cylinder material.

## Figures and Tables

**Table 1:** Properties of silica aerogels samples mentioned in Ref. [20].

Density (kg/m^3^)	Porosity (%)	Mean Pore Size (nm)	Particle Size (nm)
120	95.54	81.9	7.1
218	90.09	43.0	7.1
288	86.91	32.1	7.3
307	86.05	29.5	7.2
395	82.05	21.0	6.9
470	78.64	19.2	7.8

**Table 2:** Materials and corresponding properties [3].

Material	Density (kg/m^3^)	Thermal Conductivity under Ambient Conditions (W/(m·K))
Silica aerogel	110	0.011
SiC	3100	83.6
Carbon	1450	4.18
TiO_2_	4260	4.26
SiO_2_ fiber	2200	1.34

**Table 3:** Strengths and weaknesses of different fiber reinforcement strategies.

Strategy	Strengths and Weaknesses
I. SiO_2_ fibers (*D*_f_ = 6.0 μm) only	Increasing the volume fraction of fibers could reduce the ETC of the silica aerogels, and the ETC is the highest among these three strategies
II. SiO_2_ fibers (*D*_f_ = 6.0 μm) and SiC particles	Increasing the volume fraction of fibers would increase the ETC of the silica aerogels, the ETC is lower than Strategy I
III. SiO_2_ fibers (*D*_f_ = 3.0 μm) and SiC particles	Increasing the volume fraction of fibers would increase the ETC of the silica aerogels, the ETC is lower than Strategy I, and for higher temperatures, the ETC is lower than Strategy II

## Data Availability

All data and materials are available on request from the corresponding author. The data are not publicly available due to ongoing research using a part of the data.

## Nomenclature

*D*_f_	Diameter of reinforced fibers (m)
*D*_op_	Diameter of opacifier particles (m)
*n*_ref_	Refractive index
P	Gas pressure (Pa)
*Q*_abs_	Absorption efficiency
*Q*_ext_	Extinction efficiency
*Q*_sca_	Scattering efficiency
*T*	Temperature (K)
*f*	Volume fraction
*q*	Heat flux (W·m^−2^)
*x*	Size parameter of particles and fibers
Greek symbols	
*β*	Spectral extinction coefficient (m^−1^)
*γ*	Specific heat ratio; length ratio
*σ*	Stefan-Boltzmann constant (W·m^−2^ ·K^−4^)
*λ*	Thermal conductivity (W·m^−1^·K^−1^); Wave length (m)
*ω*	Scattering albedo
*ϕ*	Incident angle of infrared light
Subscript	
a	Pure silica aerogel
a-o	Silica aerogel and opacifier particle
b	Solid skeleton
cd	Conductive heat transfer
eff	Effective
ext	Extinction
f	Reinforced fiber
g	Gas
g-c	Gas contributed
op	Opacifier particles
r	Radiation
ref	Refraction
sca	Scattering
